# Quick tips for creating effective and impactful biological pathways using the Systems Biology Graphical Notation

**DOI:** 10.1371/journal.pcbi.1005740

**Published:** 2018-02-15

**Authors:** Vasundra Touré, Nicolas Le Novère, Dagmar Waltemath, Olaf Wolkenhauer

**Affiliations:** 1 Department of Systems Biology and Bioinformatics, University of Rostock, Rostock, Germany; 2 Babraham Institute, Babraham Research Campus, Cambridge, United Kingdom; 3 Stellenbosch Institute for Advanced Study (STIAS), Wallenberg Research Centre, Stellenbosch, South Africa; Genome Quebec, CANADA

## Introduction

With the rise of Systems Biology, the research focus moved from studying a single biological object to studying an ensemble of objects in interaction. This ensemble can be described as a network delineating the biological aspects of a data item or as a map visually representing the network’s structure and behaviour [1]. Maps have proven useful to understand biological networks, and they are frequently drawn to visually support scientific facts in presentations and journal publications. Visual representations of biological facts thus play an important role in science communication, particularly for the interdisciplinary dialogue between experimental and theoretical groups. The comprehension of such maps in a journal publication relies either on lengthy legends or, more often, on the reader’s interpretation. This interpretation is largely based on context, prior knowledge, and assumptions about the intentions of the map designer. Common symbols help to unambiguously communicate facts.

The Systems Biology Graphical Notation (SBGN, [2]) is an international, established, and widely used standard to reduce the ambiguity in representations of biological maps. The community standard provides sets of well-defined symbols, each of them with a specific biological meaning. For example, a round-corner rectangle in an SBGN map represents a macromolecule. The SBGN offers the following three complementary languages to visually describe the biology: SBGN Process Descriptions (SBGN PD [11]), SBGN Entity Relationships (SBGN ER [12]), and SBGN Activity Flows (SBGN AF [13]). Metabolic maps depicting detailed biochemical reactions, state transitions, and transport are best represented with SBGN PD. Nonmechanistic influences between biological entities, such as signaling pathways and regulatory networks, are best highlighted in SBGN AF. Finally, SBGN ER visualises independent interactions between biological entities without any temporal aspect. Maps in SBGN ER are therefore useful to avoid combinatorial explosions resulting from multicomponent complexes and molecules with multiple states.

SBGN is supported by a range of visualisation tools, including CellDesigner [5], SBGN-Editor (SBGN-ED) [6], PathVisio [7] and SBGNViz [8]. SBGN diagrams are published in open repositories such as BioModels [9] (for computational models) and Reactome [10] (for pathway data). To help scientists understand SBGN maps, the SBGN community provides detailed specification documents, software libraries [3], and online learning materials (http://sbgn.github.io/sbgn/). The benefits of having a network represented in SBGN can be summarised as: providing consistent syntax and unambiguous semantics of your visual representation [3, 4]; improving shareability, reusability, and reproducibility of your network [2]; and enabling conversion of a visual network into an executable mathematical model [5].

The creation of expressive and impactful SBGN maps requires some exercise. In the following, we share our experiences with creating SBGN maps. We offer guidelines and hints to scientists who wish to beautify and export their biological networks in SBGN. When reading the tips, please keep in mind that SBGN is a flexible standard. The provided tips should be considered as recommendations—your map will not be wrong if you do not follow them, but hopefully it will gain more impact.

## Guidelines

### Tip 1. Know the message your network should convey

The scientific question you want to address should be clear, as should the message you want to communicate to the reader. Specifying your message will help you choose what to omit, what to represent, and how to represent it. For example, when representing a network, you should think about the type of pathway you want to model: Do you want to say that phosphoinositide 3-Kinase (PI3K) activates protein kinase B (AKT, signaling information), or do you want to say that PI3K transforms phosphatidylinositol 4,5-bisphosphate (PIP2) into phosphatidylinositol (3,4,5)-trisphosphate (PIP3) and that PIP3 binds to AKT (metabolic information)? The understanding of the network’s biochemical and biophysical organisation will help you structure the map, and SBGN helps you to sketch it out.

### Tip 2. Know your audience

Keep your target audience in mind: different readers perceive different messages and focus on different aspects of a network. For example, a biochemist will generally be interested in a detailed map highlighting biological entities (e.g., macromolecules depicted in an SBGN PD map) and their interactions; whereas a cancer researcher will be concerned about mechanisms and feedback loops that occur in a specific cell type (depicted in SBGN AF notation). To ensure the successful transmission of your message, identify your audience and adapt the network's representation accordingly. Ask yourself: What do they know and what do they not know? What are they interested in? This will lead you to determine the most appropriate level of representation to visualise your network.

### Tip 3. Choose the right SBGN language

**Fig 1:**
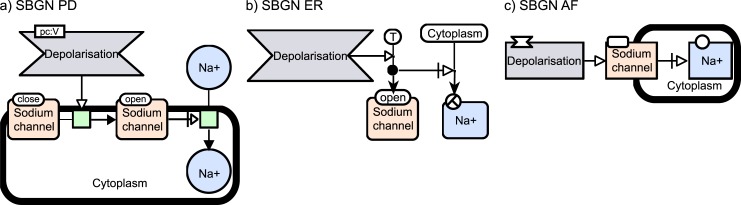
Three different SBGN representations of Na+ transport into the cytoplasm by voltage-dependant channels. (a), The depolarisation (“perturbation” symbol) of the membrane triggers (“stimulation” symbol, arrow with white head) the opening of the Na+ channel, which enables the import of Na+ from the extracellular space to the cytoplasm. Each sodium channel glyph contains a “unit of information” glyph (rectangle at the top left) that gives information about the conformational state of the channel. (b), The depolarisation stimulates the assignment of value “true” to the state “open” of the Na+ channel. This value is necessary to stimulate (“necessary stimulation” symbol, arrow with white head and a vertical line) the relocation of Na+ into the cytoplasm. (c), A cascade of signals is represented where the depolarisation stimulates the activity of Na+ channels, which in turn, is necessary to stimulate the activity of Na+ in the cytoplasm. Na+, sodium ion; SBGN, Systems Biology Graphical Notation.

Design your map in a reasonable level of detail. Fig 1 shows one biological system represented with glyphs from the three SBGN languages. In SBGN PD, we represent the fact that the depolarisation of the membrane triggers the opening of the sodium ion (Na+) channel, which enables the import of Na+ from the extracellular space to the cytoplasm. In SBGN ER, the depolarisation stimulates the assignment of value “true” to the state “open” of the Na+ channel, necessary to stimulate the relocation of Na+ into the cytoplasm. In SBGN AF, a cascade of signals is shown where the depolarisation stimulates the activity of Na+ channels, which in turn, is necessary to stimulate the activity of Na+ in the cytoplasm. For more details about the symbols used, please refer to the SBGN reference cards (http://sbgn.github.io/sbgn/templates). Deciding what knowledge to transport through your map will guide you in choosing the right SBGN flavour.

### Tip 4. Define components and interactions in the network

List the reactions constituting your network and carefully choose the names of the biological components that will be displayed as labels in the SBGN nodes. The suitable SBGN glyph to represent a biological component can be chosen by using either the reference cards (http://sbgn.github.io/sbgn/templates), the information provided by your SBGN-compliant software, or the information given in the language specifications. For example, if your component is a protein, you may use the SBGN macromolecule glyph depicted on the reference card for SBGN PD. Once your biological components are mapped on SBGN glyphs, you should choose appropriate arcs to link the components together and build the connectivity of your network.

### Tip 5. Select the right level of granularity for your map

**Fig 2:**
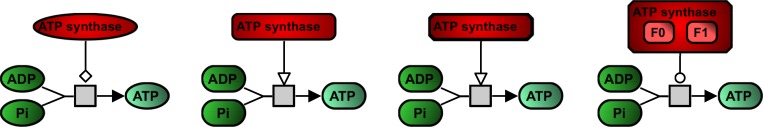
Representation of energy storage by the ATP synthase at different levels of detail from the more abstract layer (left) to the more detailed layer (right). In the first diagram, the nature of the entities is unspecified (oval shaped glyphs) and the modulation is of unknown direction. The second diagram is more detailed with a macromolecule “ATP synthase” that stimulates the reaction consuming the simple chemicals “ADP” and “Pi” to produce the simple chemical “ATP.” In the third diagram, the ATP glyph has been substituted by a complex, making the diagram even more precise. Finally, the forth diagram highlights an identified complex catalysing the synthesis of a simple chemical. ADP, adenosine diphosphate; Pi, inorganic phosphate.

A network can be visualised at different levels of detail, as exemplified in Fig 2.

The SBGN does not tell you how to represent a system, but tells your readers how to interpret what you have drawn. It is important to be as specific as you can be about the encoded biology without diluting your message. In particular, omitted information might make it hard for others to interpret your network and hence, to understand your message. At the same time, the right level of abstraction must be chosen for the important parts of your network to stand out to the readers.

### Tip 6. Design your SBGN map

A careful design of your SBGN map could provide additional biological information on top of your network. Start by creating the components and their interactions (as mentioned in Tip 4), then add necessary information in respect to Tip 5.

A good layout improves the readability of your network and can also speed up interpretation, in particular if you followed Tip 2. For instance, people working on signaling pathways generally put the plasma membrane on the top of a map and the nucleus on the bottom, with the main flow of information going downward. You can either apply an automatic layout provided by the software tool, or create the layout manually. A manual layout may be time-consuming, but the result will highlight your message better (Tip 1). Often, a combination of a an automatic layout with a manual enhancement leads to the most satisfying results.

### Tip 7. Beautify your SBGN map

**Fig 3:**
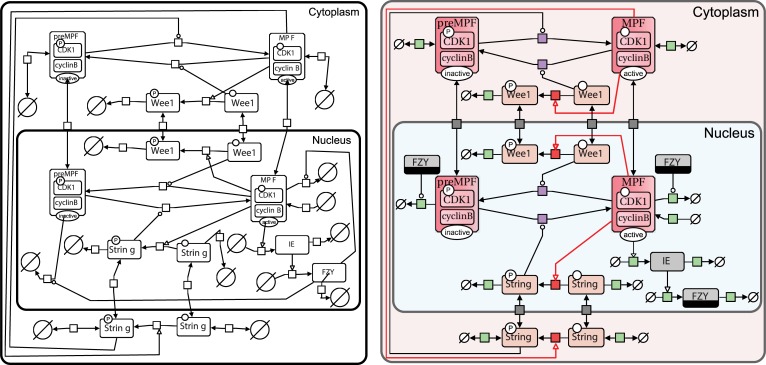
Example of the *Drosophila* cell cycle initial SBGN drawing (left) and beautified SBGN network (right). We generated the SBGN map of the following study “Dynamical modeling of syncytial mitotic cycles in *Drosophila* embryos” by Calzone et al. [16, 17]. First, we created an initial map with the different reactions found in the *Drosophila* cell cycle. Then we beautified the map by duplicating FZY with clone markers in order to reduce edge crossings, adding colors, and optimising arc positions. The colors categorise the different reactions: green processes represent creation or degradation of entities (“source and sink”) and grey processes show import and export of entities between compartments. The red processes and arcs visualise the positive feedback exerted by the MPF. The purple processes highlight the switch between the activation and the inactivation of MPF. FZY, fizzy; MPF, maturation promoting factor; SBGN, Systems Biology Graphical Notation.

Our eye catches and our brain focuses on things we find attractive. Therefore, your map should be visually appealing to your audience if you expect others to look into the details of your work. Although such graphical characteristics are neither covered nor regulated by the SBGN, add carefully chosen colors, adapt the label fonts and the size of the symbol to the components, and keep arc lengths short, etc. To limit the number of crossing arcs, make use of clone markers to duplicate “hub nodes.” This will help break up the pathway and clear the visualisation, as shown in Fig 3. Beautify your network so people want to look at it!

### Tip 8. Manage your SBGN map and its content

When satisfied with the map you designed, save it in different formats. To be able to edit the map in the future, save it in the richest source format recognised by the software (e.g., Inkscape Scalable Vector Graphics (SVG) for Inkscape, or SBML with CellDesigner extension for CellDesigner). To ensure that your readers can view your map as you designed it, a raster file format (an image made up of pixels) such as Portable Network Graphics (PNG) is probably the best, or a vector format such as Portable Document Format (PDF) for a zoomable image. Finally, save the formal representation of your map in the SBGN Markup Language (SBGN-ML) format to increase compatibility between editor and viewer software (see S1 Supporting information for the SBGN-ML files of the figures). SBGN-ML is an Extensible Markup Language (XML)-based format specifically designed to store and exchange SBGN maps [3]. By providing your map in several formats, your work will be shareable and reusable by machines and by humans.

### Tip 9. Link the original data to your SBGN map

When publishing your data, provide the SBGN visualisations saved in formats as described in Tip 8, and link them to your original data, whether the data is a model, a publication, a data set, or other. It will benefit your work and help the readers to understand your network. You can, for instance, build a Computational Modeling in Biology Network (COMBINE) archive, a container format that facilitates the sharing and reproducibility of Systems Biology projects [14]. By using the web-based CombineArchive tool [15], you can easily and quickly create a COMBINE archive by uploading and packing all files relevant to the study, among which the newly generated SBGN file.

### Tip 10. Seek help from the SBGN community

The SBGN editorial board provides and maintains guidelines, tutorials, and examples on their homepage (see S1 Table for a list of useful links). Feedback can be requested and questions can be asked on the SBGN mailing lists for end-users and developers (http://sbgn.github.io/sbgn/contact). The SBGN community is an extremely diverse and well-established community of computational biologists, modelers, computer scientists, and scientists from related fields. There will always be people willing to help, and questions are in general answered quickly. Remember, if you asked yourself a question, someone probably wondered the same before, so don’t be shy!

## Conclusions

A structured, standardised, and eye-catching map tremendously improves the readability and the reusability of your work. Communication will be more effective and your message will be passed successfully. Please consider providing your next network in SBGN!

## Supporting information

S1 Table SBGN online resources. This table provides a list of links that are useful for working with the SBGN standard. SBGN, Systems Biology Graphical Notation.
(XLSX)

S1 Supporting information Compressed SBGN-ML documents. This compressed zip file contains the SBGN-ML documents generated for the three figures presented in the quick tips. SBGN-ML, Systems Biology Graphical Notation Markup Language.
(ZIP)
